# Case Report: A Novel Synonymous ARPC1B Gene Mutation Causes a Syndrome of Combined Immunodeficiency, Asthma, and Allergy With Significant Intrafamilial Clinical Heterogeneity

**DOI:** 10.3389/fimmu.2021.634313

**Published:** 2021-02-19

**Authors:** Ioanna Papadatou, Nikolaos Marinakis, Evanthia Botsa, Marianna Tzanoudaki, Maria Kanariou, Irene Orfanou, Christina Kanaka-Gantenbein, Joanne Traeger-Synodinos, Vana Spoulou

**Affiliations:** ^1^First Department of Paediatrics, Medical School, “Aghia Sophia” Children's Hospital, National and Kapodistrian University of Athens, Athens, Greece; ^2^Immunobiology and Vaccinology Research Lab, First Department of Paediatrics, Medical School, National and Kapodistrian University of Athens, Athens, Greece; ^3^University Research Institute of Maternal and Child Health and Precision Medicine, “Aghia Sophia” Children's Hospital, National and Kapodistrian University of Athens, Athens, Greece; ^4^Laboratory of Medical Genetics, “Aghia Sophia” Children's Hospital, National and Kapodistrian University of Athens, Athens, Greece; ^5^Department of Immunology and Histocompatibility, Specialized Center and Referral Center for Primary Immunodeficiencies, Paediatric Immunology, “Aghia Sophia” Children's Hospital, Athens, Greece

**Keywords:** immunodeficiency, allergy, inborn errors of immunity, combined immune deficiency, Arpc1b, case report

## Abstract

Recently, a novel syndrome of combined immune deficiency, infections, allergy, and inflammation has been attributed to mutations in the gene encoding actin-related protein 2/3 complex subunit 1B (ARPC1B), which is a key molecule driving the dynamics of the cytoskeleton. Homozygous mutations in the ARPC1B gene have been found to result in the disruption of the protein structure and cause an autosomal recessive syndrome of combined immune deficiency, impaired T-cell migration and proliferation, increased levels of immunoglobulin E (IgE) and immunoglobulin A (IgA), and thrombocytopenia. To date, only a few individuals have been diagnosed with the ARPC1B deficiency syndrome worldwide. In this case series, we report the wide spectrum of phenotype in 3 siblings of a consanguineous family from Afghanistan with a novel homozygous synonymous pathogenic variant c.783G>A, p. (Ala261Ala) of the ARPC1B gene that causes a similar syndrome but no thrombocytopenia. Targeted RNA studies demonstrated that the variant affects the splicing process of mRNA, resulting in a marked reduction of the levels of primary (normal) RNA transcript of the ARPC1B gene in the affected patients and likely premature termination from the abnormally spliced mRNA. The next generation sequencing (NGS) studies facilitated the diagnosis of this rare combined immunodeficiency and led to the decision to treat the affected patients with hematopoietic cell transplant (HCT) from an human leukocyte antigen (HLA)-matched healthy sibling.

## Introduction

Human inborn errors of immunity (IEI) are caused by dominant or recessive, autosomal or X-linked, monogenic germline mutations causing increased susceptibility to infections, as well as a growing diversity of autoimmune, autoinflammatory, allergic, and/or malignant phenotypes (1).

The introduction of next generation sequencing (NGS) in the early 2010s has revolutionized the diagnosis of inborn errors of immunity, facilitating the identification of numerous novel genes associated with primary immunodeficiency, autoimmunity, and atopy (2, 3).

Recently, a novel syndrome of combined immune deficiency, allergy, and inflammation has been attributed to mutations in the gene called actin-related protein 2/3 complex subunit 1B (ARPC1B) (4). Briefly, human actin-related protein complex, which is essential for actin filament branching and is a key molecule driving the dynamics of the cytoskeleton (5), has two ARPC1 component isoforms, with ARPC1B prominently expressed in blood cells. Mutations in the ARPC1B gene have been found to result in the disruption of the protein structure and cause an autosomal recessive syndrome of combined immune deficiency, impaired T-cell migration and proliferation, increased levels of immunoglobulin E (IgE) and immunoglobulin A (IgA), and thrombocytopenia (5). To date, only a few individuals have been diagnosed with the ARPC1B deficiency syndrome worldwide (4, 5).

Because of its recent discovery and extreme rarity, the exact mechanisms and the full spectrum of the severity of the disease remain unclear. In a previous case series of 14 patients from different parts of the world, several disease-causing variants were identified (Supplementary Figure 2) throughout the ARPC1B gene, and the patients demonstrated a variety of disease spectrum and severity, including recurrent bacterial and viral infections, eczema, food allergies, asthma, and thrombocytopenia (4). Therefore, it is not clear whether there is a direct genotype–phenotype relationship or if there are other factors determining disease presentation.

Here, we describe the wide phenotypic spectrum in three siblings of a consanguineous family from Afghanistan with a homozygous synonymous pathogenic variant c.783G>A, p. (Ala261Ala) of the ARBC1B gene, as demonstrated by whole exome sequencing (WES) studies. Targeted RNA studies demonstrated that the variant affects the splicing process of mRNA predicted to result in reduced levels of the primary (normal) transcript of the gene and premature termination following transcription of the abnormally spliced product.

## Case Description

### Index Patient (II-6)

The family tree is depicted in Supplementary Figure 1. The index patient (II-6) is a male child of consanguineous parents from rural Afghanistan. He was presented at our tertiary pediatric center in Athens, Greece, at 6 months of age in critical condition due to a severe lower respiratory tract infection (LRTI) with wheezing and difficulty in breathing. He had been residing with his family in the refugee camp of Lesvos island for 2 months prior to his emergency air transfer, during which time he required five brief hospital admissions for LRTIs with significant leukocytosis (WBC20.000–48.000).

He was born in Turkey by uneventful normal vaginal delivery; the umbilical cord was detached within the first 2 weeks of life, and he was an exclusively breast-fed infant. As described by his mother at presentation, he has a 14-year-old brother with “dry skin, recurrent infections, and hypothyroidism (II-1),” a 10-year-old sister with “delayed growth” (II-3), and two other phenotypically healthy siblings (II-4 and II-5). The second child of the family (II-2) had died at 3 years of age due to an unidentified infection of the central nervous system (CNS).

Upon examination, the patient appeared tachypneic and tachycardic, with significantly diminished breath sounds in the left (L) lung and wheezing in the right (R) lung. Imaging revealed a large opacity occupying nearly the entire left lung, and blood tests demonstrated elevated white blood cells (WBCs) (41.720/μl, Neu 78%) and c-reactive protein levels (136 mg/l). He was treated with IV teicoplanin and piperacillin-tazobactam, nebulizers, and supplemental oxygen, and he gradually improved over the next 3 weeks. Two days before the planned discharge, he developed a perianal abscess due to *Pseudomonas aeruginosa* and became, again, systemically unwell. While under treatment with IV ceftazidime and clindamycin, he developed a second severe LRTI on Day 30 of hospitalization. On Day 35, he developed an anaphylactic shock after having received formula milk for the first time. He then gradually developed severe eczema over the next 2 weeks. II-6 also failed to thrive (body weight <3rd percentile, growth parallel to the 3rd percentile) (Table 1).

**Table 1:** Clinical parameters of the three patients with the ARPC1B mutation.

**Patient**	**Age^#^**	**Infections**	**Allergy**	**Autoimmunity**	**Other**
II-6	6 months	Recurrent bronchiolitis, pneumonia, and skin abscess	Eczema, food allergy, and allergic asthma	Autoimmune hypothyroidism	Failure to thrive
II-1	14 years	Recurrent pneumonia, recurrent lymphadenitis, and skin abscesses	Eczema	Autoimmune hypothyroidism	Failure to thrive
II-3	10 years	Recurrent viral upper respiratory tract infections	/	Autoimmune hypothyroidism	Failure to thrive, iron-deficiency anemia

# *t presentation*.

In view of the recurrent severe infections and allergic symptoms, extensive immunological work-up was performed. Findings included elevated IgE and IgA, as well as normal levels of complement factors C3, C4, and CH50. Specific antibodies to routine vaccine antigens were not tested due to uncertain immunization history and lack of documentation. The patient is A Rh (+), and anti-group B haemagglutinins were positive. Flow cytometry analysis of peripheral blood leucocytes revealed normal counts of total B (*CD20*), T (*CD3*), and NK (*CD3–CD56/CD16*+*)* cells, a reversed ratio of helper CD4+/cytotoxic CD8+ T-cells, as well as reduced numbers of naïve CD4+ and CD8+ T-cells with a reversed ratio of naïve/memory T cells. T-helper 17 (Th17) count and the scores of the dihydrorhodamine (DHR) flow cytometric test, as well as interleukin-1 receptor-associated kinase 4 (IRAK-4) assays, were normal; T-cell proliferation assays also yielded normal results. Specific IgE were at high class (method: ImmunoCap) for multiple food allergens (Table 2).

**Table 2:** Immunological Parameters of the 3 patients with the ARPC1b mutation at presentation.

**Patient**	WBC^*^ (Neu)	**IgE**	**IgG**	**IgA**	**IgM**	**CD3**	**CD19**	**CD4**	**CD8**	**NK**	**CD4naïve/memory**	PLTs^*^ (MPV)
	**Cells/μL (%)**	**IU/ml (Normal range for age)**	**mg/dl (Normal range for age)**			**cells/μL**					**Ratio**	**Cells/μL (fL)**
II-6	18.140 (29)	**279** (1–60)	561 (316–1,148)	**140** (13–69)	200 (47–204)	**1,446**	3,892	1,312	134	162	**36/64**	427.000 (8.0)
II-1	14.520 (80)	**11,642** (8–309)	**2,300** (955–1,995)	**1,620** (85–214)	63 (60–372)	2,578	719	1,679	861	137	**8/92**	337.000 (7.6)
II-3	10.940 (50)	**2,942** (3–562)	1,550 (891–2,042)	**617** (52–331)	109 (63–275)	2,362	676	910	1,146	288	**8/91**	228.000 (7.6)

*Immunoglobulin classes were measured in serum. The concentration of IgE is in IU/mL, and IgG, IgA, and IgM in mg/dL. The concentration of lymphocytes subpopulations is presented as × 10^9^ cells/L. WBC, white blood cells; CD3, T lymphocytes; CD19, B lymphocytes; CD4, helper T cells; CD8, cytotoxic T cells; NK, natural killer cells; PLTs, platelets; MPV, mean platelet volume; IgE, immunoglobulin E; IgG, immunoglobulin G; IgA, immunoglobulin A; IgM, immunoglobulin M. Bold indicates deviation from normal values for age*.

* *during a well visit*.

The phenotype of patient II-6 was highly indicative of autosomal recessive combined immunodeficiency; therefore, a WES was ordered with a special focus on previously described combined immune deficiency (CID) genes. A homozygous mutation in the ARPC1B gene was found, consistent with the CID phenotype of the patient.

### Patient II-1

The eldest son of the family, aged 14 years (II-1), presented with severe eczema, bilaterally enlarged cervical lymph nodes, exophthalmos, tinea capitis, and failure to thrive (<3rd percentile for height). With regard to his past medical history, recurrent skin and respiratory tract infections, as well as hypothyroidism, were described. Shortly after his first assessment in the clinic, he presented with an acute severe pneumonia, and he was admitted for further management. Infectious diseases work-up was negative for tuberculosis (IGRA and gastric aspirates) and a panel of viral infections (multiplex PCR). Blood cultures were negative, but the patient responded well to empiric antibiotic therapy. His immunological work-up yielded similar results to his youngest brother (II-6), in addition to very high levels of IgE for age and the presence of anti-thyroid autoantibodies, as shown in detail in Table 2. Targeted genetic testing for ARPC1B showed the same homozygous mutation as II-6.

### Patient II-3

The 10-year-old daughter (II-3) of the family presented with significant failure to thrive, height, and weight well-below the third percentile for age and gender. Her skin was normal, and there was no history of asthma, autoimmunity, or recurrent severe infections. Her immunological work-up yielded similar results to II-1 and II-6, in addition to significant microcytic anemia, as shown in detail in Table 2. Targeted genetic testing for ARPC1B showed the same homozygous mutation as II-1 and II-6.

**Figure 1:**
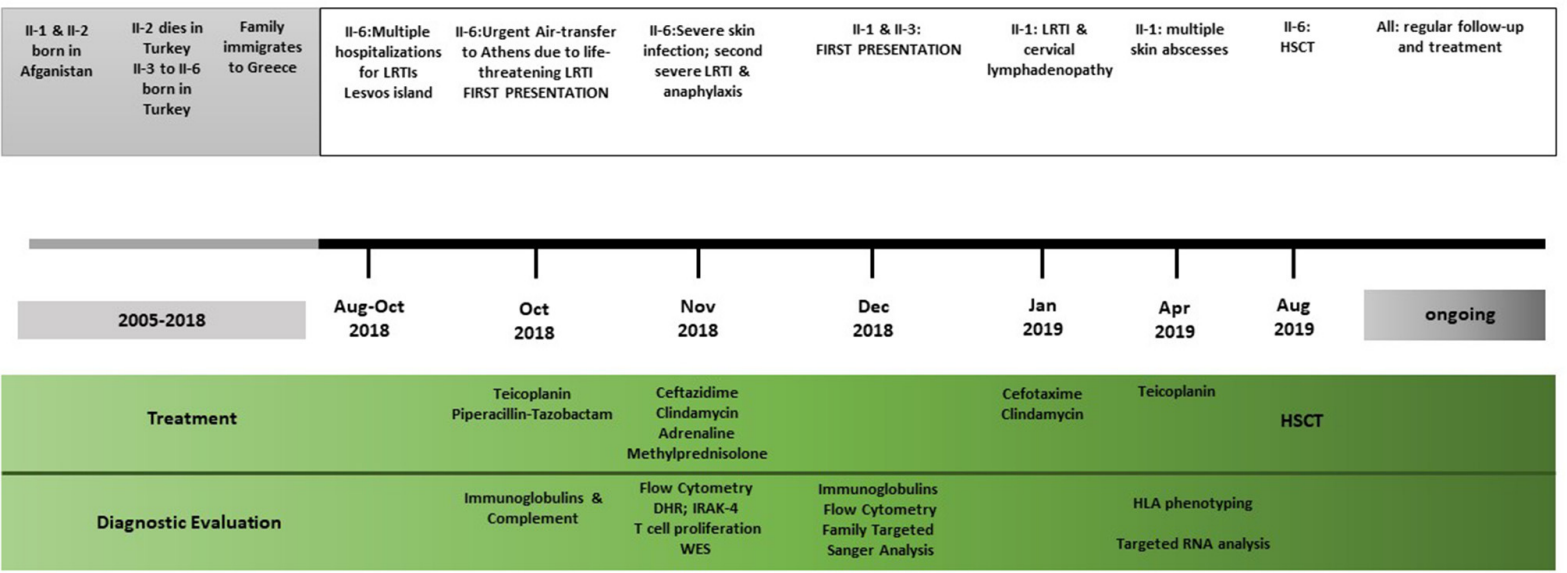
Timeline of the clinical events, diagnostic evaluations, and treatment strategies. LRTI, lower respiratory tract infections; HSCT, haematopoietic stem cell transplant; DHR, dihydrorhodamine flow cytometric test; IRAK, interleukin-1 receptor-associated kinase 4; WES, whole exome sequencing.

The other two children of the family, a 7-year-old boy (II-4) and a 5-year-old girl (II-5), were fit and healthy and were found to be growing normally along the 25th percentile for both height and weight. They were phenotypically healthy, and genetic testing showed that they did not carry any mutated copy of the ARPC1B gene. Timeline of clinical presentation and management is summarized in Figure 1.

## Diagnostic Assessment, Follow-Up, and Outcomes

### Genetic Testing: Whole Exome Sequencing, Targeted Sanger Sequencing, and Targeted RNA Studies

The diagnostic strategy involved WES analysis of genomic DNA (gDNA) from the proband (II-6), followed by targeted analysis using Sanger sequencing of DNA samples from the proband, the parents, and the other family members, to confirm the findings and support the evaluation of segregation for likely candidate variant identified. Targeted ARPC1B RNA analysis of WBCs from the proband (II-6), his father (I-2), his sister (II-5), and healthy controls was performed to study the effect of the candidate variant in mRNA splicing and protein synthesis. The expression of ARPC1B in WBCs made it easily accessible for RNA studies. The parents provided written informed consent for all individuals included in this study prior to clinical WES, Sanger sequencing, and RNA analysis. Bioinformatic analysis was conducted using SOPHiA DDM platform by Sophia Genetics, VarAFT application (6), and VarSome system (7).

**Figure 2:**
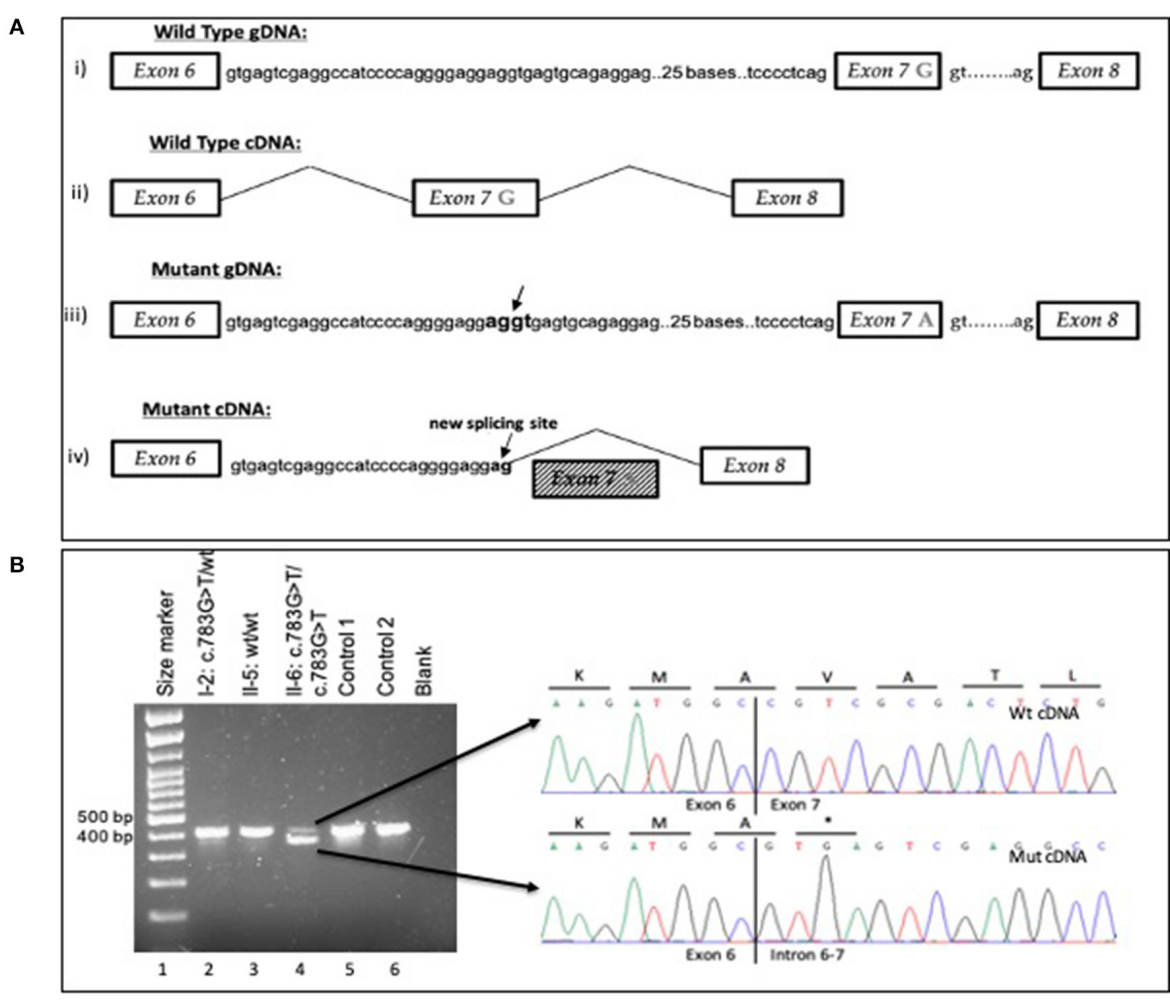
**(A)** Schematic overview of the wild-type and mutant gDNA and cDNA of exons 6–8 in the ARPC1B gene (i) Wt gDNA with G nucleotide at the last position of exon 7 (ii) Wt cDNA from mRNA analysis (iii) Mutant gDNA with A to G at the last position of exon 7 Arrows represent cryptic splice site in intron 6–7 with 124 nucleotides upstream from the variant G783A (iv) Mutant cDNA represents the result of variant c.783G>A; exon 7 skipping and partly intron 6–7 retention (29 bp) and the activation of a cryptic splice site with 124 nucleotides upstream in intron 6–7. The using transcript for gDNA and cDNA analysis was ENST00000252725.9. **(B)** Agarose gel electrophoresis for cDNA analysis for variant c.783G>A in ARPC1B. Reverse transcription (RT)-PCR showed leaky splicing with a weak wild-type band with an expected size of 450 bp and a stronger band of around 400 bp, yielded by the skipping of exon 7 of the ARPC1B gene, for patient II-6 (lane 4). For the heterozygous father I-2 (lane 2), RT–PCR showed a stronger wild-type band and a weaker band for the exon 7 skipped product. For the wild-type homozygous II-5 (lane 3), Control 1 (lane 5), and Control 2 (lane 6) enabled a strong wild-type band. Sequencing of the different cDNA products showed unaltered splicing for the smaller product (upper electropherogram) and skipping of exon 7 of the ARPC1B gene (electropherogram below) for the larger product, leading to a premature stop codon on the protein level. Underline letters represent the amino acids and * the stop codon.

In the proband (II-6), a homozygous ARPC1B variant NM_005720.4:c.783G>A, located in the last nucleotide position of exon 7, was identified through the analysis of the WES data. At the protein level, there is no amino acid change in p. (Ala261Ala). However, the nucleotide substitution was predicted to affect splicing. The variant is recorded in dbSNP database (rs779597975) but without information of pathogenicity or protein effect. The variant was confirmed by Sanger sequencing in the II-6 gDNA sample of the proband. Segregation analysis by Sanger sequencing on the gDNA samples of his family members confirmed the autosomal recessive inheritance of the variant and the phenotype of the other members (Supplementary Figure 1). Moreover, the alteration of the wild-type donor splice site was predicted to affect splicing by the Human Splicing Finder tool (8). The variant was predicted to affect pre-mRNA splicing and its likely use of a cryptic splice site. Sanger sequencing of the cDNA sample of the patient revealed that the c.783G>A variant causes the loss of a wild-type donor splice site at the last nucleotide position in exon 7 of ARPC1B. This variant consequently activates a cryptic splice site containing 124 nucleotides upstream in intron 6–7. These findings revealed exon 7 skipping and partial retention of a 29 bp sequence of intron 6–7 (29 bp) (Figure 2A). In contrast, Sanger sequencing of cDNA samples from WBCs of healthy controls did not reveal any alteration in the splicing mechanism. The sequencing analysis of the products of the cDNA analysis indicated that the variant creates a stop codon and, therefore, a truncated 236 amino acid protein NP_005711.1:p. (Val237*) (Figure 2B). A small amount of wild-type mRNA was detected. Based on these findings and according to guidelines from the American College of Medical Genetics (ACMG) (9), the variant was classified as pathogenic (PVS1, PM2, PP1, PP3, and PP4).

### Follow-Up and Management

Following discharge, patient II-6 was started on preventive asthma therapy with daily oral Montelukast and nebulized fluticasone and chemoprophylaxis against infection with trimethoprim/sulfamethoxazole. Emergency IM adrenaline pen was also prescribed. Patient II-1 was also put on chemoprophylaxis with trimethoprim/sulfamethoxazole, a hypoallergic diet, and topical agents for eczema. A high-caloric dietary plan was made for patient II-3, in addition to oral iron supplementation. All 3 patients were scheduled for regular follow-up appointments with a multidisciplinary team of pediatrics, immunology, infectious diseases, and bone marrow transplant. In view of his severe phenotype and unstable condition, patient II-6 successfully received a hematopoietic cell transplant (HCT) following myeloablative conditioning at 12 months of age. The donor was his 7-year-old healthy brother (II-4), who was a 10/10 HLA-matched donor. Complete donor-derived hematopoietic chimerism has consistently been detected after Day +60 after transplantation. Currently, more than 1 year after the HCT, the patient is in excellent clinical condition with no signs of graft vs. host disease, no skin abnormalities, and sufficient growth and gain of weight.

Patient II-1 has suffered several serious infections since the diagnosis was made 14 months ago, including generalized lymphadenitis, skin abscesses, parotic gland abscess, and LRTIs necessitating hospitalizations for IV antibiotics. He has also lost weight despite having an age-appropriate diet. In view of his rapid deterioration, he is also scheduled to receive haematopoietic stem cell transplant (HSCT) from his HLA-matched healthy 5-year-old sister (II-5).

Patient II-3 remains under regular follow-up in a stable condition.

## Discussion

Actin-related protein 2/3 complex subunit 1B (ARPC1B) deficiency is a newly characterized syndrome of combined immune deficiency, allergy, and autoimmunity that affects multiple organs due to the crucial role of ARPC1B in the regulation of actin polymerization and the stability of the cytoskeleton (10).

Till date, only a small number of patients with ARPC1B deficiency syndrome caused by a number of different mutations in the ARPC1B gene have been reported worldwide, presenting with a wide spectrum of disease complexity and severity (4, 11).

In this study, we report 3 siblings whose combined immune deficiency with infections and allergic phenomena and autoimmunity is caused by a synonymous homozygous variant c.783G>A in ARPC1B, which has not been previously described.

Similar to cases with ARPC1B variants described previously (4), our patients suffered from recurrent infections, allergic reactions, asthma, autoimmunity, and failure to thrive. Several groups have explored how ARPC1B mutations affect immune cells and lead to a broad immune dysregulation syndrome including susceptibility to infection, allergy, autoimmunity, and autoinflammatory processes. The ARPC1B gene is a member of the Arp2/3 complex through which actin cytoskeletal dynamics are regulated. Actin and actin-regulating proteins have long been recognized as key components of the cytoskeleton that control diverse processes of the immune system, including cellular infrastructure, cellular motility, cell signaling, and vesicle transport (12). The involvement of ARPC1B in the nucleation of branched actin filaments makes it critical to the formation of the immune synapse in T cells and in the endocytosis and phagocytosis of Ag presenting cells (13). Defects in cytoskeleton rearrangement, altered immunologic synapses formation, and reduced chemotaxis have been identified in the T cells of patients with ARPC1B deficiency, neutrophils, and NK cells. Brigida et al. recently showed that ARPC1B deficiency was associated with the inability of T cells to extend an actin-rich lamellipodia upon T-cell receptor (TCR) stimulation and to assemble an immunological synapse. ARPC1B-deficient T cells additionally displayed impaired TCR-mediated proliferation and SDF1-α-directed migration (14, 15). Arp2/3 function has also been reported to be critical for the formation of the T-regulatory (Treg) cell function. The defective Treg cell function is suggested to be involved in both the exaggerated T_H_2 responses and IgE reactivity against allergens seen in ARPC1B deficiency. Kuijpers et al. have shown that ARPC1B deficiency impairs neutrophil motility and chemotaxis due to an F-actin polymerization defect, which may be the basis of susceptibility to bacterial infections in the presence of normal antibody levels and in part explain the variable inflammatory responses seen in patients with ARPC1B deficiency. Similarly, NK cells of ARPC1B-deficient patients show migration defects and NK-cell dysfunction, which may contribute to the predisposition to viral infections (4).

In regards to the pathogenesis of the combined immune deficiency phenotype of the three patients described in this study, *in silico* analysis have shown that the variant c.783G>A of the ARPC1B changes the wild-type donor site and was predicted to be causative in the splicing process. Although synonymous variants are generally considered as non-pathogenic, they have been reported to affect pre-mRNA splicing and alter protein translation. Further investigation of the donor splice site c.783G>A variant was detected by the WES aimed to elucidate the pathogenicity of this variant based on the cDNA analysis of the ARPC1B gene transcript. The data obtained on the cDNA of patient II-5 showed skipping of the variant lead to exon 7 and partial retention of intron 6–7 (29 bp). In addition, the variant activates a cryptic 5′ donor splice site with 124 nucleotides upstream from the pathogenic site. During the splicing process of the mutant pre-mRNA, the cryptic splice site on intron 6–7 and the normal acceptor splice site in intron 7–8 were utilized. These disorders cause disruption of the ARPC1B protein functions, including cytoskeleton rearrangement, formation of immunologic synapses, and chemotaxis, leading to the increased susceptibility to infection, autoimmunity, and allergy seen in our 3 patients. Most importantly, the cDNA analysis revealed that the major mRNA isoform was the mutant, but there was also a wild-type mRNA isoform. The presence of different isoforms of mRNA, that produce isoforms of protein, including wild-type protein, could justify the various expressivity and heterogeneity on the phenotype of the patient. However, cDNA–RNA studies are restrained to relative quantifications and could not correspond exactly to the clinical heterogeneity presented in the family. The actual amount of normal protein seems to be the key factor of the heterogeneity; however, protein studies that are more appropriate to evaluate the noticed differences are unfortunately not available in our setting. Moreover, the possible impact of additional genetic or other environmental and epigenetic factors on the clinical heterogeneity cannot be excluded.

Most interestingly, our patients did not demonstrate any platelet dysfunction or coagulation pathology. This is in contrast with the latest International Union of Immunological Societies (IUIS) classification of the inborn errors of immunity, where ARPC1B deficiency is classified as a combined immune deficiency with congenital thrombocytopenia, along with the Wiskott–Aldrich syndrome protein-interacting protein (WIP) deficiency (1). This classification of ARPC1B deficiency is based on a previously published case series where the majority of patients had bleeding tendency due to thrombocytopenia, and 79% of patients suffered from recurrent or severe bleeding episodes, mostly from the gastrointestinal tract (4). Such bleeding disorders in patients with ARPC1B deficiency have been associated with mild platelet dysfunction and aggregation defect, slight reduction of CD62P and CD63 molecules (15), as well as defects in platelets size and morphology with the reduction of calcium-rich platelets dense granules and the inability to form lamellipodia necessary for actin branching (5). However, the 3 patients mentioned in the study have no history of bleeding episodes and had normal platelet counts with normal volume at all timepoints. Aggregation as measured by a platelet function assay (PFA) test with collagen/epinephrine was normal.

Differences in disease phenotype among patients with genetic diseases could be attributed either to the distinct mutations of a gene resulting in different outcomes or to variable expressivity of the disease, through other genetic and/or environmental factors (16). With respect to the findings in this study, since our patients carry a novel mutation of the gene that has not been described before, this mutation may indeed seemingly have little or no effect on platelet cells. At the same time, an identical genotype has resulted in different disease spectrum and severity in the three affected siblings, suggesting variable expressivity. Based on these findings, we hypothesize that the homozygous donor splice site c.783G>A variant in the ARPC1B gene almost completely abolishes the production of the primary mRNA transcript from the gene in the patients, and the abnormally spliced transcript is predicted to undergo premature termination of translation p. (Val237*). In conclusion, we have identified a novel mutation of the ARPC1B gene, which leads to combined immune deficiency with recurrent skin and respiratory infections, allergic reactions, asthma, and autoimmunity but not bleeding disorders. We are very grateful to the patients, their family, and all the multidisciplinary health professionals involved in their care.

## Patient Perspectives

Index patient II-6 1 year after HSCT is leading a normal life at his family home, growing well, and with no further major infections or hospitalizations. Patient II-1 is eager to undergo the scheduled HSCT with the hope to overcome most of his chronic conditions. Patient II-3 is happy and thriving on supportive care.

## Data Availability Statement

The name of the repository and accession number can be found at: Sequence Read Archive (SRA) by NCBI, accession number PRJNA690210.

## Ethics Statement

Written informed consent was obtained from the individual(s), and minor(s)' legal guardian/next of kin, for the publication of any potentially identifiable images or data included in this article.

## Author Contributions

IP is one of the primary physicians of the patients and drafted the manuscript. NM conducted the genetic testing and drafted the manuscript with equal contribution to IP. EB and IO were the primary physicians of the patients and reviewed the manuscript. MT and MK conducted the immunology work-up and offered immunological consultation. JT-S supervised the genetic work-up, offered genetic consultation, and reviewed the manuscript. CK-G and VS lead the multidisciplinary care of the patients and reviewed the manuscript. All authors contributed to the article and approved the submitted version.

## Conflict of Interest

The authors declare that the research was conducted in the absence of any commercial or financial relationships that could be construed as a potential conflict of interest.
