# Supplementary material for: Case Report: A Novel Synonymous ARPC1B Gene Mutation Causes a Syndrome of Combined Immunodeficiency, Asthma, and Allergy With Significant Intrafamilial Clinical Heterogeneity
Source: Front Immunol. 2021 Feb 19;12:634313. doi: 10.3389/fimmu.2021.634313 (PMC7933039; doi:10.3389/fimmu.2021.634313)
Supplement: Supplementary file 1 [file Image_1.pdf]

Supplementary Figure 1:

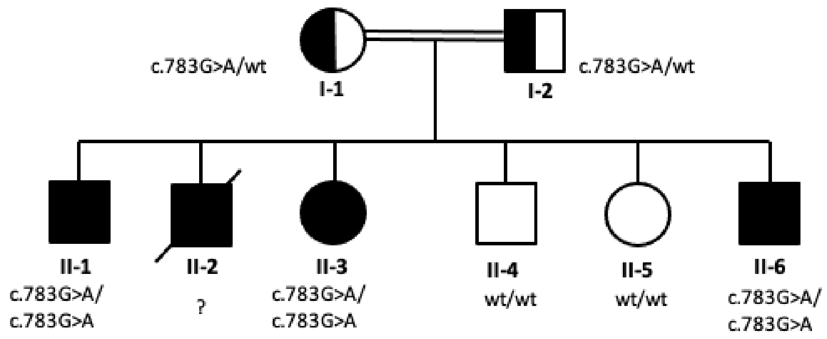

Family pedigree with the zygosity of the pathogenic variant per member. Solid symbols represent the affected probands (diagonal line shows death). Half solid symbols represent unaffected relatives, which are carriers for the variant. Open symbols represent unaffected relatives.
