# Supplementary material for: Case Report: A Novel Synonymous ARPC1B Gene Mutation Causes a Syndrome of Combined Immunodeficiency, Asthma, and Allergy With Significant Intrafamilial Clinical Heterogeneity
Source: Front Immunol. 2021 Feb 19;12:634313. doi: 10.3389/fimmu.2021.634313 (PMC7933039; doi:10.3389/fimmu.2021.634313)
Supplement: Supplementary file 2 [file Image_2.pdf]

Supplementary figure 2:

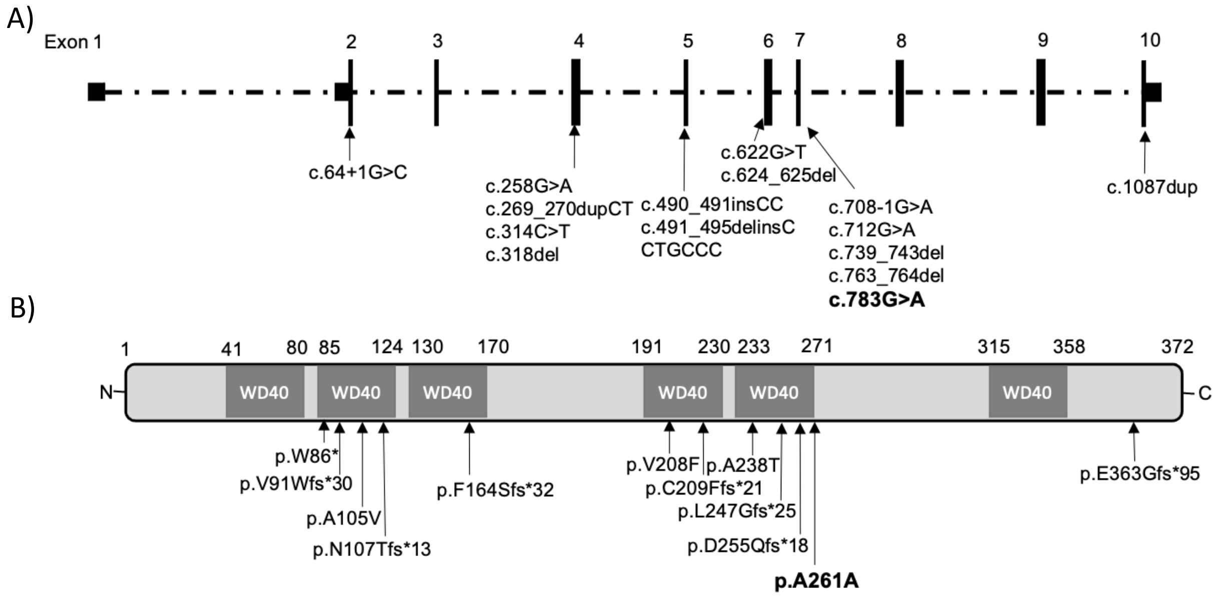

A) Nucleotide positions of identified causative variants relative to ARPC1B coding exons (NM_005720.3). Two splice site, 3 missense, 1 nonsense and 8 frameshift variants have been identified until today. Variant in bold is the identified variant in present study. B) ARPC1B has 6 WD40 repeat domains forming a β-propeller required for Arp2/3 complex function. At the protein level there is no amino-acid change, however the sequencing analysis of the cDNA analysis products indicated that the variant creates a stop codon and therefore a truncated 236 amino acid protein NP_005711.1:p.(Val237*).
